# Impact of Neck and Shoulder Pain on Health-Related Quality of Life in a Middle-Aged Community-Living Population

**DOI:** 10.1155/2021/6674264

**Published:** 2021-06-08

**Authors:** Masaaki Machino, Kei Ando, Kazuyoshi Kobayashi, Hiroaki Nakashima, Masayoshi Morozumi, Shunsuke Kanbara, Sadayuki Ito, Taro Inoue, Hiroyuki Koshimizu, Taisuke Seki, Shinya Ishizuka, Yasuhiko Takegami, Yukiharu Hasegawa, Shiro Imagama

**Affiliations:** ^1^Department of Orthopedic Surgery, Nagoya University Graduate School of Medicine, Japan; ^2^Department of Orthopedic Surgery, Kariya Toyota General Hospital, Japan; ^3^Department of Rehabilitation, Kansai University of Welfare Sciences, Japan

## Abstract

**Purpose:**

Neck and shoulder pain (NSP) is very common in the general population. However, scarce information exists on the relationship between NSP and health-related quality of life (HRQOL) outcomes in this population. The present study described NSP prevalence and its impact on the HRQOL of middle-aged and older persons undergoing a routine medical checkup.

**Methods:**

This study recruited 318 subjects (125 males and 193 females; average age, 63.4 years) in good health, collected underwent anthropometric measurements, physical function examinations, and blood testing. This study defined NSP as the presence of muscle tension, stiffness, pressure, or dull pain in areas between the neck and the arch of the scapular. Study subjects were divided into two groups (NSP (+) and NSP (−) groups). The subjects completed questions on the Medical Outcomes Study 36-item short-form health survey (SF-36) and the EuroQol 5-dimension, 5-level version (EQ-5D-5L) tool.

**Results:**

Of the patients, 150 and 168 were NSP (+) and NSP (−), respectively. The NSP complaint rate was 47.2%. The NSP (+) group had younger and more female participants than the NSP (−) group. In the multivariate regression analysis, the NSP (+) group had lower physical QOL based on the SF-36 physical component summary (odds ratio (OR), 2.45) and lower mental QOL based on the SF-36 mental component summary (OR, 2.05). Overall, the NSP (+) group had a higher risk of having low QOL scores (EQ-5D-5L index; OR, 1.76).

**Conclusions:**

The NSP (+) rate in healthy middle-aged and older persons was 47.2%. Furthermore, NSP (+) status was directly related poor HRQOL. NSP is a predictor of suboptimal physical and mental QOL. Therefore, NSP prevention or intervention for NSP may improve middle-aged and older adults' QOL.

## 1. Introduction

Neck and shoulder pain (NSP), which has a prevalence ranging from 16% to 75%, contributes to musculoskeletal disability that influences an individual's physical, social, and psychological well-being and a society's effect on domestic and socioeconomic status [1, 2]. In the general population, people commonly complain of NSP. Previous studies describe NSP as either neck pain, nonspecific neck pain, or chronic NSP among other terms [3–10].

Two-thirds of the European and North American populations complain of neck pain in their lifetime [11]. Multicenter studies have documented the prevalence of neck pain and stiffness among healthy volunteers using the visual analog scale (VAS) [12, 13]. In their research study, Hurwitz et al. focused on neck pain between upper regions of the thoracic spine to the occiput and surrounding musculature regions [6]. Additionally, Bliss et al. reported that almost all maladies concerning regions superior to the shoulders present with neck pain [14].

Health-related quality of life (HRQOL) indicators provide useful disease information to research, clinicians, and health planner [15, 16]. HRQOL instruments are commonly used to extensively assess a community's health [17]. However, a few reports are available investigating the influences of NSP on the HRQOL in the healthy general population. This study described the NSP prevalence's impact on HRQOL indicators among middle-aged and older adults who presented for routine examination.

## 2. Materials and Methods

### 2.1. Study Population

Study participants were recruited from volunteers undergoing routine medical assessments funded by Yakumo's local administration in 2019. In Yakumo, Japan, where approximately 17,000 live, 28% of them are aged over 65 years and the majority are involved in the agricultural and fishing-related activities. An HRQOL survey is administered as well [18–21]. Blood samples were obtained, anthropometric measurements were conducted, and physical functions were assessed. The participants underwent these evaluations after overnight fasting on the basis of previous study [22].

Patients with prior documented surgery to the limbs and spine, serious injury to their knee(s), with severe osteoarthritis, prior hip or spine fracture, disorders of nervous system, severe mental disease, diabetes, renal or cardiac disease, and severe movement or standing disabilities or any disorder of the central or peripheral nervous system were excluded from this study. Participants that had not fasted prior to their check-ups were also ineligible for inclusion. Of the 537 potential study participants, 318 (125 males and 193 females) were eligible for inclusion in this study [23]. The study protocol was approved by Nagoya University Graduate School of Medicine's human research ethics' committee and institutional review board (IRB approval no: 2014-0207). Written informed consent was obtained from all individuals before study participation. The study protocol adhered to the principles of the Declaration of Helsinki.

### 2.2. Study Definitions

**Figure 1:**
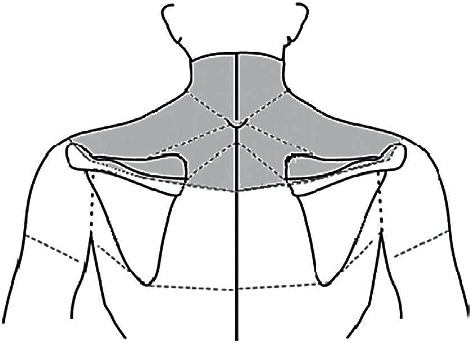
Participants' NSP location. A schematic of NSP location as documented by Takasawa et al.

We defined NSP as the presence of muscle tension, stiffness, pressure, or dull pain in regions extending from an individual's neck to his or her scapular arch [9]. The NSP localization is shown in Figure 1 [13]. No participant had neck and shoulder surgery. Participants with possible neck ailments (e.g., cervical spondylotic radiculopathy and cervical disc herniation) were ineligible to participate in this study. The participants were firstly asked whether they had NSP in the one month preceding the interview date [13]. Moreover, study subjects were categorized into two groups NSP (+) and NSP (−). Additionally, several variables were compared. The participants then described their pain intensity using the VAS as *no pain* (a score of 0) and *pain as bad as it could be* or *worst imaginable pain* (a score of 100 on a 100 mm scale) [24]. As done in our previous studies, VAS was used to describe the prevalence of low back pain (LBP) and knee joint pain (KJP) [25, 26].

### 2.3. Anthropometric Measurements

We used bioelectrical impedance analysis (BIA) to collect anthropometric information including body weight, body mass index (BMI), percent body fat (PBF), appendicular skeletal muscle index (aSMI) representing muscle mass, and neck circumference (NC). A BIA unit, the InBody 770 body composition and body water analyzer (InBody, Seoul, Republic of Korea), distinguished tissues (such as fat, muscle, and bone) based on their electrical impedance [27]. Previous studies have established the accuracy of BIA measurements [28]. Each participant grasped the analyzer's handles in which electrodes were embedded and rested each foot on platform similarly embedded with two electrodes. BMI was computed by dividing a participant's body weight in kilograms by the square of his or her height in meters (m^2^). The PBF was computed by dividing the fat mass in kilograms by the body weight measured in kilograms and multiplying the obtained result by 100. The aSMI was calculated to document the arm and leg skeletal muscle; this was done by dividing a subject's mass in kilograms by the square of his or her measured body height in meters (m^2^) [29]. NC was computed by the InBody 770 BIA device [27]. With a participant standing with his or her head positioned in the Frankfort horizontal plane and the shoulders relaxed, NC was measured from a level below the laryngeal prominence perpendicular to the long axis of the neck using a nonstretchable plastic tape. We documented all results in centimeters by rounding off all measures [30, 31].

### 2.4. Physical Performance

Grip strength was measured using the Toei Light Handgrip Dynamometer (Toei Light Co., Saitama, Japan) [32]. Both hands were simultaneously tested with subjects in a standing position. An average value was deemed a participant's grip strength. The back muscle strength (i.e., the trunk muscles' maximal isometric strength) was measured in a standing position with 30° of lumbar flexion using a digital back muscle strength meter (T.K.K.5402; Takei Scientific Instruments Co., Niigata, Japan) [26, 27]. Participants performed two tasks to evaluate mobility. Participants were first asked to quickly walk for 10 meters in a straight line at their fastest pace; task completion time was taken to be a participant's 10 m gait time [32]. Participants were then asked to rise from a sited position on a standard chair 46 cm high and walk 3 m, turn around, walk back to the same chair, and sit down. This process was repeated, and the average time was documented as the result of the 3 m timed up-and-go test (3 m TUG) [26, 27].

### 2.5. Blood Tests

An autoanalyzer (JCA-RX20; Nihon Denshi, Tokyo, Japan) was used to conduct biochemical analyses on blood samples [27].

### 2.6. The HRQOL

The subjects completed the Medical Outcomes Study 36-item short-form health survey (SF-36) [32, 33] and the Japanese version of the EuroQol 5-dimension, 5-level version (EQ-5D-5L) [34].

The SF-36 (Japanese ver. 2.0) was used to evaluate subjects' QOL [33]. Participants individually completed all questionnaires unless they required assistance. These questions assessed the SF-36's eight scales and two summary measures, the physical component summary (PCS) and the mental component summary (MCS). A PCS < 50 and MCS < 50 were considered poor physical and mental QOL, respectively [32].

The EQ-5D-5L is a self-administered tool listing five dimensions including mobility, self-care, usual activities, pain/discomfort, and anxiety/depression. Each dimension's severity is rated as either no problem, a slight problem, moderate problems, severe problems, or extreme problems [35]. The results of each of the five dimensions are summarized into a five-digit number that signifies a health status, e.g., *11111* implying the absence of problems and *55555* suggesting the existence of extreme problems. So far, this summary has defined 3,125 health states that can be transformed to a solitary health index by weighting each response. The Japanese version of the EQ-5D-5L value was used to obtain the EQ-5D-5L index. This index had been assessed using the EuroQol Group's crosswalk methodology [36, 37]. This study defined poor QOL as an EQ-5D-5L index < 0.875 [34].

### 2.7. Statistical Analysis

We used the SPSS statistical software (version 25.0; SPSS Statistics, IBM Corp., Armonk, NY, USA) to analyze our data. Continuous variables were summarized as the means and standard deviations (SDs) and categorical variables as proportions. The chi-square and the Mann–Whitney *U* tests were used to describe between-group differences. Factors attaining a *p* value of <0.05, and age and gender as confounders, were entered into a multivariate logistic regression model to describe predictors of poor HRQOL. Subsequently, we described prevalence odds ratios (ORs) and their corresponding 95% confidence intervals (CI). Throughout, a *p* value of <0.05 was considered significant.

## 3. Results

The mean age, BMI, and PBF of the 318 study participants were 63.4 years (range, 40–87 years; SD, 10.0 years), 23.7 kg/m^2^, and 29.4%, respectively. The prevalence of NSP in all participants was 47.2% (150 of 318). Moreover, the NSP prevalence was higher in females (108 of 193) than in males (42 of 125) (56.0% vs. 33.6%, *p* = 0.0002).

Of the subjects, 150 and 168 were NSP (+) and NSP (−), respectively. In addition, the prevalence of NSP was 47.2%. The mean VAS of NSP was 35 ± 25 mm (range, 10–80 mm) in the NSP (+) group.  Table 1 shows the comparative data between the NSP (+) and NSP (−) groups by gender. The NSP (+) participants were younger than the NSP (−); furthermore, there were more females among the NSP (+) participants than in the NSP (−) group. The NSP (+) group had shorter body height, higher PBF, lower aSMI, and lower grip strength compared with the NSP (−) group. No significant difference was confirmed in the NC (manual and BIA) and blood test results between the two groups (Table 1).

**Figure 2:**
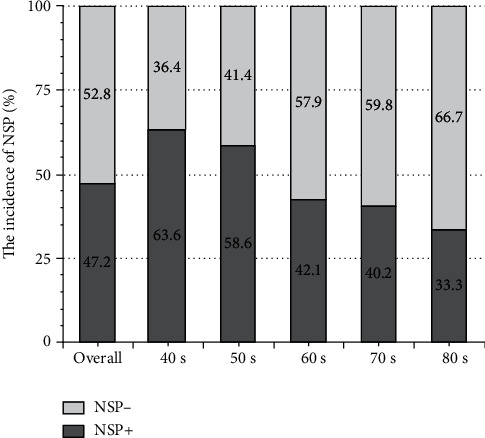
The incidence of NSP by the age group. The highest NSP incidence was among subjects in their 40s and 50s. Hereafter, the NSP incidence decreased with increasing age.

Among subjects in their 40s, 50s, 60s, 70s, and 80s, the prevalence of NSP was 63.6% (21 of 33), 58.6% (41 of 70), 42.1% (48 of 114), 40.2% (37 of 92), and 33.3% (3 of 9), respectively; NSP prevalence was inversely related to age. Participants aged ≤50 years had the highest NSP prevalence. The analysis also showed that more than 50% of the middle-aged participants had NSP (Figure 2).

The NSP (+) group had lower SF-36 values in all the eight assessed domains. However, no significant difference in emotional role and mental health was noted between the two groups. The PCS of the SF-36 was lower in the NSP (+) group than in the NSP (−) group for both men and women. However, the MCS was lower in the NSP (+) group only for women (Table 2).

Both men and women in the NSP (+) group had a low EQ-5D-5L index. In each EQ-5D-5L dimension, the NSP (+) group had higher scores in all items except for anxiety/depression. On the other hand, the NSP (+) group had higher pain/discomfort dimension scores in both men and women than in the NSP (−) group (Table 3).

The multivariate logistic regression analysis, which included age and sex as confounding factors, illustrated that the prevalence of NSP was associated with poor HRQOL according to SF-36 and EQ-5D-5L. The NSP prevalence was a predictor for a low physical QOL score (PCS < 50; OR, 2.451; *p* = 0.001). In addition, the NSP prevalence was the only predictor for a low mental QOL score (MCS < 50; OR, 2.047; *p* = 0.007) and low QOL (EQ-5D-5L index, <0.875; OR, 1.761; *p* =0.017). On the other hand, body height, PBF, aSMI, and grip strength were not significantly related to poor HRQOL (Table 4).

## 4. Discussion

There still is controversy regarding NSP definition, like the heterogeneous definitions in the literature [3–10]. The broad concept that Takasawa et al. defined that was more accurate was used to enroll and assess NSP participants [13]. Recent studies document impaired trapezius muscle pump function and poor trapezius muscle perfusion and oxygenation in times of psychophysiological stress and when doing repetitive work. These studies also illustrated lesser pain thresholds especially in the superior portion of the trapezius muscle among persons with NSP [8, 38]. These findings suggest that the trapezius muscle is involved in NSP pathogenesis NSP.

We investigated the relationship NC and presarcopenia in a previous study [23]. A decreased NC, based on BIA assessments, was directly related to having presarcopenia. In the current study, we focused on NSP's impact on the HRQOL and presented a different aspect using a population previously analyzed by ourselves. In the literature, NSP prevalence rises to its peak in middle age and subsequently decreases [7, 8]. This study revealed that more than 50% of participants in their 40s and 50s experienced NSP and NSP prevalence decreased with age. Men and women have dissimilar NSP prevalence. NSP is more prevalent in working woman populations and individual women in the general community [7, 8]. This is because women have lower pressure and pain thresholds in their trapezius muscles when compared to men, based on reports by Binderup et al. [38]. Additionally, women experience more stress and have more concerns than men. The mental stress experienced by women negatively impacts on the trapezius' muscle hemoglobin function [39].

This study is the first to reveal the impact of NSP on the HRQOL in middle-aged and older persons during a routine medical examination. The EQ-5D-5L is a simple, efficient standardized, and validated tool that can evaluate five general health profiles and how they influence the EQ-5D-5L index [34]. Our study's EQ-5D-5L index implies that having NSP increases the likelihood of experiencing a poorer overall HRQOL. We also found in EQ-5D-5L dimensions that NSP has more influence on the physical QOL than the mental QOL. Hence, NSP pharmacologic or rehabilitation therapy may improve the QOL of middle-aged and older adults.

The EQ-5D index of NSP (+) individuals (0.86) is higher in persons with diabetes (0.80), people living with HIV (PLHIV) (0.80), those with dermatological diseases (0.73), respiratory diseases (0.66), dengue fever (0.66), frail older persons (0.58), older persons following a fall (0.46), and those with fracture (0.23) [40–44].

There were some limitations. First, study participants who were drawn from a single center were healthy middle-aged and older adults of a single race living in a relatively rural setting and engaged in agricultural or fishing occupations. Thus, they may not be representative of the general population [27, 28]. Future research should employ longitudinal approaches in urban areas. Second, the cross-sectional design employed limits causal inferences between NSP and HRQOL. Nonetheless, the present study that enrolled healthy adults provides an insight into NSP. Furthermore, the relationship between NSP and HRQOL illustrated by this could aide in NSP management. Therefore, health workers should intervene upon recognizing NSP is in healthy middle-aged and older persons during routine checkups to improve their QOL.

## 5. Conclusion

The prevalence of NSP was 47.2% in healthy middle-aged and older adults, and NSP was associated with poor HRQOL. Therefore, preventing or intervening in NSP may improve middle-aged and older adults' QOL.

## Figures and Tables

**Table 1:** Comparison between with and without neck and shoulder pain according to sex.

Variables	Total			Male			Female		
	NSP+	NSP−	*p*	NSP+	NSP−	*p*	NSP+	NSP−	*p*
Number of subjects	150	168		42	83		108	85	
Age (years)	61.4 ± 10.5	65.2 ± 9.21	*.0012*	64.0 ± 10.3	67.0 ± 8.45	.1608	60.4 ± 10.4	63.4 ± 9.62	*.0345*
Sex (male/female)	42/108	83/85	*.0002*						
Body height (cm)	157.4 ± 8.06	160.1 ± 8.60	*.0051*	165.0 ± 6.10	166.0 ± 6.95	.4038	154.4 ± 6.62	154.4 ± 5.75	.9989
Body weight (kg)	59.8 ± 11.7	60.5 ± 11.6	.6064	67.7 ± 10.3	67.0 ± 9.79	.9729	56.7 ± 10.8	54.1 ± 9.66	.0855
BMI (kg/m^2^)	24.0 ± 3.82	23.5 ± 3.44	.1990	24.9 ± 3.10	24.3 ± 2.83	.4691	23.7 ± 4.04	22.7 ± 3.80	.0866
PBF (%)	31.4 ± 7.79	27.7 ± 6.90	*<.0001*	24.6 ± 4.08	23.4 ± 4.28	.2037	34.0 ± 7.29	31.8 ± 6.42	*.0304*
aSMI (kg/m^2^)	6.64 ± 0.94	6.97 ± 1.12	*.0068*	7.70 ± 0.60	7.79 ± 0.76	.4304	6.21 ± 0.67	6.16 ± 0.77	.3208
NC by manual (cm)	34.9 ± 3.09	35.4 ± 3.40	.1303	38.0 ± 2.19	38.0 ± 2.43	.8383	33.8 ± 2.54	32.9 ± 2.07	*.0076*
NC by BIA (cm)	33.9 ± 3.222	34.3 ± 3.41	.2312	36.9 ± 2.05	36.7 ± 2.27	.7837	32.9 ± 2.07	31.9 ± 2.60	*.0074*
Grip strength (kg)	27.2 ± 8.48	30.2 ± 9.29	*.0067*	37.9 ± 6.17	37.5 ± 6.88	.6006	23.0 ± 4.72	23.3 ± 5.00	.3547
Back muscle strength (kg)	78.8 ± 65.5	87.7 ± 34.4	.1735	112.2 ± 24.6	113.3 ± 26.9	.8335	65.5 ± 17.8	61.8 ± 17.6	.3240
10 m gait time (s)	5.03 ± 0.83	4.98 ± 0.94	.4342	4.73 ± 0.83	4.77 ± 0.77	.8734	5.15 ± 0.80	5.20 ± 1.06	.8896
TUG (s)	5.96 ± 1.01	5.99 ± 1.10	.9137	5.62 ± 0.98	5.79 ± 1.05	.4560	6.10 ± 0.99	6.20 ± 1.12	.5784
Albumin (g/dL)	4.35 ± 0.24	4.37 ± 0.23	.3469	4.36 ± 0.28	4.37 ± 0.26	.6296	4.35 ± 0.23	4.36 ± 0.19	.4949
Total cholesterol (mg/dL)	211.0 ± 32.5	203.4 ± 34.9	.1264	202.3 ± 28.8	192.8 ± 35.1	.1919	214.4 ± 33.3	213.5 ± 31.8	.9679
Triglycerides (mg/dL)	94.1 ± 61.7	91.8 ± 49.3	.8730	93.5 ± 44.6	101.1 ± 59.0	.9932	94.4 ± 67.4	83.1 ± 35.9	.6297
CRP (mg/dL)	0.12 ± 0.34	0.10 ± 0.36	.5985	0.17 ± 0.45	0.15 ± 0.51	.8629	0.11 ± 0.28	0.06 ± 0.07	.1093
Prevalence of LBP (%)	54, 36.0%	43, 25.6%	.0588	13, 31.0%	19, 22.9%	.4482	41, 38.0%	24, 28.2%	.2055
Prevalence of KJP (%)	30, 20.0%	26, 15.5%	.3629	4, 9.5%	10, 12.0%	.9025	26, 24.1%	16, 18.8%	.4827

The values are given as the mean and the standard deviation (mean ± SD). Italic values indicate significant difference. NSP: neck and shoulder pain; BMI: body mass index; PBF: percent body fat; aSMI: appendicular skeletal muscle index; NC: neck circumference; BIA: bioelectrical impedance analysis; TUG: timed up-and-go; CRP: C-reactive protein; LBP: low back pain; KJP: knee join pain.

**Table 2:** Impact of neck and shoulder pain status on SF-36 according to sex.

Variables	Total			Male			Female		
	NSP+	NSP−	*p*	NSP+	NSP−	*p*	NSP+	NSP−	*p*
PF	85.2 ± 17.5	89.5 ± 13.3	*.0067*	86.8 ± 19.5	90.0 ± 12.9	.1703	84.5 ± 16.6	89.1 ± 13.7	*.0212*
RP	87.6 ± 17.3	90.4 ± 18.4	*.0499*	89.3 ± 14.7	90.9 ± 19.8	.3056	86.9 ± 18.2	89.9 ± 16.9	.1251
BP	61.7 ± 20.4	75.1 ± 22.5	*<.0001*	62.0 ± 22.3	76.1 ± 23.4	*.0010*	61.5 ± 19.7	74.1 ± 21.8	*<.0001*
GH	65.0 ± 17.9	70.3 ± 18.9	*.0060*	67.7 ± 18.9	71.0 ± 19.1	.1812	63.9 ± 17.5	69.6 ± 18.8	*.0176*
VT	58.4 ± 16.7	64.0 ± 15.4	*.0010*	64.6 ± 14.8	66.0 ± 15.7	.3185	55.9 ± 16.8	62.1 ± 15.1	*.0045*
SF	87.4 ± 17.2	90.6 ± 17.2	*.0379*	89.9 ± 13.2	92.2 ± 16.9	.2300	86.4 ± 18.5	89.0 ± 17.4	.1688
RE	88.7 ± 17.4	90.2 ± 18.0	.2344	90.5 ± 19.1	92.1 ± 14.1	.3091	87.4 ± 18.4	89.8 ± 17.1	.1793
MH	73.7 ± 15.8	75.9 ± 16.1	.1127	77.3 ± 16.7	77.6 ± 13.8	.4641	72.2 ± 16.3	74.4 ± 15.6	.1647
PCS	45.9 ± 11.6	50.3 ± 9.30	*<.0001*	45.9 ± 11.5	50.2 ± 9.07	*.0127*	45.8 ± 11.7	50.4 ± 9.56	*.0020*
MCS	49.9 ± 9.02	52.5 ± 8.02	*.0030*	52.1 ± 8.73	53.5 ± 7.67	.1889	48.9 ± 9.02	51.6 ± 8.28	*.0214*

The values are given as the mean and the standard deviation (mean ± SD). Italic values indicate significant difference. SF-36: 36-item short-form health survey; NSP: neck and shoulder pain; PF: physical functioning; RP: role-physical; BP: bodily pain; GH: general health perception; VT: vitality; SF: social functioning; RE: role-emotional; MH: mental health; PCS: physical component summary; MCS: mental component summary.

**Table 3:** Impact of neck and shoulder pain status on EQ-5D-5L according to sex.

Variables	Total			Male			Female		
	NSP+	NSP−	*p*	NSP+	NSP−	*p*	NSP+	NSP−	*p*
EQ-5D-5L index	0.86 ± 0.12	0.91 ± 0.11	*<.0001*	0.86 ± 0.13	0.92 ± 0.12	*.0027*	0.86 ± 0.11	0.89 ± 0.11	*.0103*
EQ-5D-5L dimensions									
Mobility	1.28 ± 0.60	1.19 ± 0.59	*.0454*	1.26 ± 0.54	1.17 ± 0.55	.1880	1.30 ± 0.63	1.20 ± 0.65	.1502
Self-care	1.09 ± 0.33	1.04 ± 0.33	*.0387*	1.12 ± 0.39	1.03 ± 0.28	.1127	1.08 ± 0.31	1.04 ± 0.47	.2102
Usual activities	1.21 ± 0.50	1.13 ± 0.40	*.0467*	1.14 ± 0.35	1.11 ± 0.41	.3230	1.25 ± 0.55	1.13 ± 0.42	*.0303*
Pain/discomfort	2.07 ± 0.82	1.65 ± 0.67	*<.0001*	2.05 ± 0.85	1.60 ± 0.66	*.0008*	2.07 ± 0.81	1.70 ± 0.67	*.0004*
Anxiety/depression	1.27 ± 0.54	1.20 ± 0.51	.1362	1.26 ± 0.70	1.17 ± 0.51	.2236	1.29 ± 0.47	1.24 ± 0.53	.2273

The values are given as the mean and the standard deviation (mean ± SD). Italic values indicate significant difference. NSP: neck and shoulder pain; EQ-5D-5L: EuroQol 5-dimension, 5-level version.

**Table 4:** Risk factors for poor HRQOL in multivariate logistic regression analysis adjusted for age and gender.

Variables	Odds ratio	95% confidence intervals	*p*
SF-36 PCS < 50			
Prevalence of NSP	2.451	1.441-4.171	.001
SF-36 MCS < 50			
Prevalence of NSP	2.047	1.221-3.433	.007
EQ-5D-5L index < 0.875			
Prevalence of NSP	1.761	1.105-2.808	.017

Only significant factors are shown. SF-36: 36-item short-form health survey; PCS: physical component summary; NSP: neck and shoulder pain; MCS; Mental Component Summary; EQ-5D-5L: EuroQol 5-dimension, 5-level version.

## Data Availability

The cohort data used to support the findings of this study are restricted by the Institutional Review Board of Nagoya University Graduate School of Medicine in order to protect the privacy of subjects in Yakumo study.
